# Chronic Diabetic Complications: Current Challenges and Opportunities

**DOI:** 10.3390/jcm11030673

**Published:** 2022-01-28

**Authors:** Ilias N. Migdalis, Leszek Czupryniak, Nebojsa Lalic, Nikolaos Papanas, Paul Valensi

**Affiliations:** 1 Diabetes Centre, Second Department of Internal Medicine, NIMTS Hospital, 11521 Athens, Greece; 2 Department of Internal Medicine and Diabetology, Medical University of Warsaw, 02-097 Warsaw, Poland; czupnniak@vahoo.co.uk; 3 Faculty of Medicine, University of Belgrade, Clinic for Endocrinology, Diabetes and Metabolic Diseases UCC of Serbia, 11000 Belgrade, Serbia; lalic.nm@gmail.com; 4 Diabetes Centre, Second Department of Internal Medicine, Democritus University of Thrace, 68132 Alexandroupolis, Greece; papanasnikos@yahoo.gr; 5 Unit of Endocrinology-Diabetology-Nutrition, AP-HP, Jean Verdier Hospital, Paris 13 University, Sorbonne Paris Cité, CRNH-IdF, CINFO, 93140 Bondy, France; paul.valensi@aphp.fr

The Special Issue, “Chronic Diabetic Complications: Current Challenges and Opportunities”, is rich in scientific content, covering a wide field of diabetic complications via both original studies and reviews. Psillas et al. [1] discuss the effects of diabetes mellitus, hypercholesterolaemia and hypertension on Bell’s palsy. Migdalis et al. [2] provide data on agents used to treat hyperglycemia, hypertension and dyslipidemia in subjects with type 2 diabetes mellitus (T2DM) and chronic kidney disease. Margaritidis et al. [3] have shown that Biphasic Insulin Aspart 30 is equally effective to premixed human insulin in controlled subjects with T2DM. Kovářová et al. [4] report that different molecular species of fetuin A influence its variable actions. Further insights into retinopathy and autonomic neuropathy are provided by Hsing et al. [5] and Kempler et al. [6], respectively. In Taiwan, Tai et al. [7] have shown discrepancies between urban and rural areas in terms of diabetic complications. In Italy, Russo et al. [8] have obtained favorable results with edoxaban in patients with atrial fibrillation and diabetes.

Gouveri and Papanas [9] and Penlioglou et al. [10] have revisited olfactory dysfunction and stroke, respectively, in diabetes. Dascalu et al. [11] have conducted a systematic review on diabetic retinopathy following bariatric surgery in T2DM. Baranowska-Jurkun et al. [12] provide an update on microvascular complications in prediabetes. Finally, Koliaki et al. [13] highlight the practical issues and challenges of diabetes management in the COVID-19 era.

Overall, a very satisfactory harvest. Diabetic complications remain a huge challenge [14,15,16]; we hope that this Special Issue will contribute to their understanding. We also sincerely thank all the authors for their contributions.
